# Characterization of Hybrid Materials Prepared by Sol-Gel Method for Biomedical Implementations. A Critical Review

**DOI:** 10.3390/ma14071788

**Published:** 2021-04-05

**Authors:** Michelina Catauro, Stefano Vecchio Ciprioti

**Affiliations:** 1 Department of Engineering, University of Campania “Luigi Vanvitelli”, Via Roma 29, I-813031 Aversa, Italy; 2 Department of Basic and Applied Science for Engineering (S.B.A.I.), Sapienza University of Rome, Via del Castro Laurenziano 7, Building RM017, I-00161 Rome, Italy

**Keywords:** sol-gel method, FT-IR spectroscopy, thermal analysis, organic–inorganic hybrids, radical scavenging capacity, antibacterial activity

## Abstract

The interaction between tissues and biomaterials (BM) has the purpose of improving and replacing anatomical parts of the human body, avoiding the occurrence of adverse reactions in the host organism. Unfortunately, the early failure of implants cannot be currently avoided, since neither a good mixture of mechanical and chemical characteristics of materials nor their biocompatibility has been yet achieved. Bioactive glasses are recognized to be a fine class of bioactive substances for good repair and replacement. BM interact with living bones through the formation of a hydroxyapatite surface layer that is analogous to bones. Bioglasses’ composition noticeably affects their biological properties, as does the synthesis method, with the best one being the versatile sol-gel technique, which includes the change of scheme from a ‘sol’ fluid into a ‘gel’. This process is widely used to prepare many materials for biomedical implants (e.g., hip and knee prostheses, heart valves, and ceramic, glassy and hybrid materials to serve as carriers for drug release). Nanoparticles prepared by the sol-gel method are interesting systems for biomedical implementations, and particularly useful for cancer therapy. This review provides many examples concerning the synthesis and characterization of the above-mentioned materials either taken from literature and from recently prepared zirconia/polyethylene glycol (PEG) hybrids, and the corresponding results are extensively discussed.

## 1. Introduction

The human body does not always manage to repair tissues and organs damaged by trauma or different diseases. In recent years, many efforts have been made to overcome these problems by developing new advanced (and performing) materials for the repair of tissues or body parts, since the premature collapse of implants is usually induced by various reasons that can largely be prevented.

The failure of many implants is the result of rejection by the patient owing to a reaction with the immune system, but can also lead to chemical, mechanical, tribological, surgical, and biocompatibility inconveniences. For instance, a dental or orthopedic patient may suffer inflammation and frequent infection processes after implantation, which can lead to unsuccessful osseointegration. Several studies have described high oxidative stresses due to inflammatory processes that may cause large formations of granulation tissue and fibrous capsule around the prostheses and an osteolysis process as a consequence [1,2]. To this end, new biomaterials have been developed [3], with porous structures that enable appreciable tolerability and integration capability, thus allowing the regeneration of bone injuries. Different classes of materials have been investigated with the aim of producing durable implants by combining appropriate chemical and mechanical properties, along with biocompatibility. In this context, bioactive glasses constitute fine materials for good repair and replacement [4,5], since they form a hydroxyapatite layer on their surfaces, which is analogous to the mineral phase of bones [6], thus bonding them with the living bone.

In 1971, Hench et al. [7] discovered the first bioglass, and since then, numerous other glass compositions have been analyzed [8]. Furthermore, several authors [9,10] have stressed that their biological properties are affected by both their composition and their synthesis method.

However, it is worth noting that the best method for preparing bioglasses is the sol-gel technique, for which the system undergoes a transition from a mostly colloidal liquid (‘sol’) into a solid ‘gel’ [11]. This method offers the possibility of preparing different materials with novel, fine-tuned properties using a simple and versatile process, which is commonly used to synthesize ceramics and glass materials at low temperatures. The purity of the products and the possibility of incorporating thermolabile molecules are among the most important advantages of the sol-gel method. In recent years, organic–inorganic hybrid materials with glasses and ceramics, and materials with nanocomposites synthesized for drug delivery [12] have been proposed as interesting biomaterials for several pharmaceutical and biomedical applications [13,14]. In the former systems, the organic and inorganic phase are bonded together at nanometer to sub-micrometer scales [15,16], while the latter incorporate polymers or drugs into the inorganic matrix [12], or nanocomposites derived by hybrid materials. The synthesized materials have applications in the orthopedic field as prostheses [17,18,19].

The bioactivity of sol-gel glasses was found to be higher than that of materials obtained by melting methods with comparable composition [20,21]. The characteristic presence of –OH groups on their surface is responsible of heir bioactivities, since they are able to induce hydroxyapatite nucleation.

Coating techniques, e.g., dip, spin or spray coating, can be coupled with the traditional sol-gel method, in order to enhance biomedical applications by modifying the surface properties of the implants, thus causing a decrease of erosion [17] and ion liberation, resulting in improved bioactivity and biocompatibility [22,23]. This approach improves the material performances through the decrease of (toxic) ion release from metallic materials that are responsible for the implant failure when they touch body fluids, preventing them from being embedded in bone [18].

Ceramic and glassy materials are among the products of this synthesis those used as carriers for drug delivery [24,25,26]. The materials used as prostheses are capable of both releasing drugs (such as anti-inflammatory agents, antibiotics, anticancer agents) and depressing the activity of inflammatory processes or infections caused by their implantation [27,28,29]. In particular, antitumor drugs contained in the sol-gel have been analyzed a lot. The non-selective action of antitumor drugs is one of the main problems faced in cancer therapy, since chemotherapy drugs do not selectively kill only cancer cells, but can also have side effects on healthy tissues and organs, often affecting both cancer cells and healthy tissues and organs.

Nanoparticles prepared using the sol-gel method by loading different amounts of antitumor drugs play a major role in innovative and more specialized cancer therapy. The size of nanoparticles, obtained by carefully controlling each synthesis parameter, plays a preeminent role in enhancing the deep penetration and local accumulation of the drug in the tumor tissues [30,31].

## 2. Innovative Process: Sol-Gel

**Figure 1:**
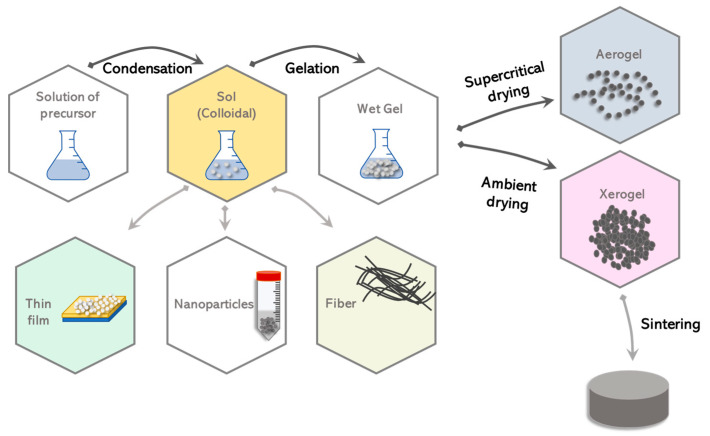
Representative image of different products obtained using sol-gel processing.

The operative conditions of the sol-gel method can be selected in order to provide materials with controlled structures and properties (Figure 1).

The sol-gel method involves inorganic polymerization chemistry. Starting from molecular precursors, an oxide is obtained via hydrolysis and polycondensation reactions.

Bulk gels, films, powers, and nanoparticles are prepared starting from the addition of the precursor to a solvent (alcohol). The formation of a sol, a stable suspension of colloidal particles in a liquid, in the initial stage occurs via the first hydrolysis reaction (Equation (1)) [11].

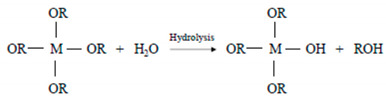
(1)

The alkoxide OR moiety in the reaction is changed by the hydroxyl group. Furthermore, condensation takes place according to Equations (2) and (3) before hydrolysis comes to an end.

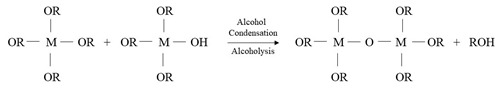
(2)

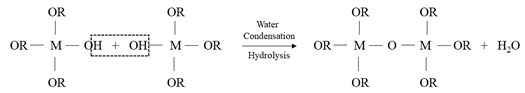
(3)

High reactivity is exhibited by metal alkoxides towards water, and this could give rise to a spontaneous process such as the formation of precipitates [32]; to avoid this drawback, reactants are added that act as catalysts.

Furthermore, several parameters may affect either the hydrolysis or condensation rates of sol-gel synthesis, thus influencing the structural properties of the thus-obtained material (i.e., the precursors, catalysts, molar ratios of reactants, solvent composition and the aging and drying conditions) [33].

The solvent is commonly used as a homogenizing agent, since water and some precursors are practically non-miscible, and because it simplifies hydrolysis. In order to decrease the hydrolysis rate, the chain length of the precursors and their branching are usually increased. Furthermore, both hydrolysis and condensation reactions are affected by the molar ratios of the reactants; indeed, the increase of this value causes the promotion of hydrolysis and increases the reaction rate, thus leading to the achievement of hydrolysis before condensation has occurred. The molar ratio of the reactants influences either the gelation time or the dimensions of the pores. As a matter of the fact, the higher the degree of aggregation and condensation, the higher the viscosity of the sol. The gelation is the achievement of a continuous network. Prior to this, the colloidal dispersion has a more or less viscous behavior. Indeed, the viscosity increases acutely, leading to the formation of an elastic-body gel. This increase in the viscosity is due to the growth of the particles during aggregation or condensation, resulting from the collisions in the sol. Thus, this leads to clusters of particles, which become bigger through repetitions of the collisions. Once there are many clusters in the sol phase, they will progressively link to the spanning clusters, forming a gel. After the occurrence of gelation, the chemical reactions are not finished, and structural rearrangements take place in the wet gels. This, denoted as aging, increases the gel stiffness.

The drying stage, which involves the removal of the liquid phase from the wet gel upon heating at normal pressure or at ambient temperature with a decrease in the external atmospheric pressure, is a tricky part of the whole sol-gel process. As evaporation occurs, even drying stresses can cause a cracking of the bulk materials. In fact, during the drying stage, a shrinkage phenomenon of the gel happens as a consequence of the liquid flowing out from the inner to the gel body surface. As drying continues, the network becomes increasingly more stiff, and the liquid surface tension increases correspondingly, because of the decrease of the pore radii. Furthermore, two different systems can be achieved using different drying conditions: a xerogel or an aerogel can be obtained at ambient pressure or in supercritical conditions, respectively (Figure 1).

## 3. Hybrid Materials

Hybrids are new materials with enhanced properties with respect to those of the original components [34,35]. Hybrid materials have the characteristic of having strictly distributed phases, and the particle size of each phase varies from 1 to 100 nm [36]. In recent decades, polymer scientists have developed new challenging routes for preparing hybrids, with the aim of combining the remarkable and versatile properties of different classes of polymers with those of inorganic components (playing as hosts) to be exploited in different applications (see Table 1).

**Figure 2:**
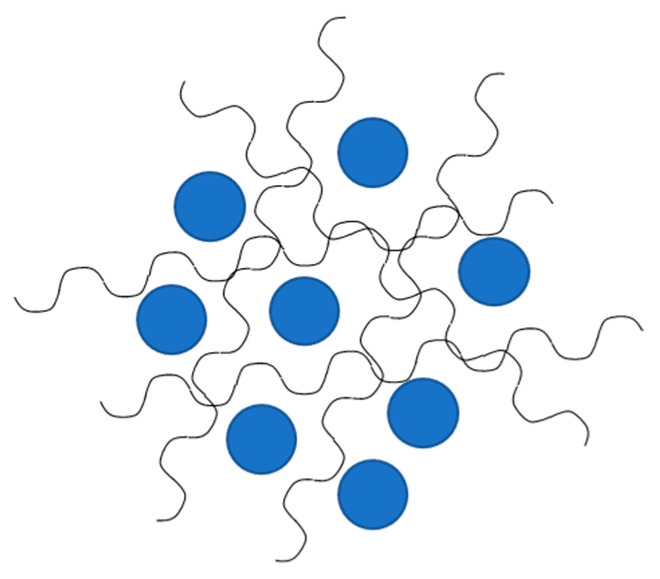
Inorganic nanoparticles in the polymer solution.

The simplest method for synthesizing a hybrid material is solution blending, where a polymer is first dissolved in a suitable solvent, while later an appropriate amount of inorganic nanoparticles are added, so as to produce a dispersion through ultrasound or mechanical stirring. This technique is quite simple and cheap, but it has disadvantages, as it favors the tendency to agglomerate the nanoparticles. The phenomenon of agglomeration of nanoparticles leads to a worsening of the properties of the materials, due to non-homogeneity with consequent poor physical, chemical and mechanical properties. To overcome this problem, inorganic nanoparticles are added to the solution after functionalization with organic molecules (Figure 2) [45].

Melt blending is a more environmentally friendly synthesis method compared to solution blending, since it does not require the use of organic solvents. Many studies in the literature describe preparations of hybrids materials with polymer according to the melt-blending technique, in which the polymers are combined with different kinds of inorganic components [45].

In the last two or three decades, the sol-gel method has been widely used for the preparation of organic–inorganic hybrids, with low processing temperature and pressure, versatility and flexibility being the main advantages over other methods [46,47]. As mentioned above, syntheses are carried out at low temperatures, and therefore the thermal decomposition of the trapped organic materials is minimized, resulting in extremely pure or porous materials [48].

Furthermore, by varying the chemistry of the precursors, it is possible to control the size of the particles and their porosity [49].

Many organic–inorganic hybrids whose inorganic component is made up of silica, alumina, titania, germania, and vanadia [50,51] have been synthesized using this technique, making it a versatile method for designing new organic–inorganic hybrid materials [52,53]. The properties of the hybrid materials include a synergy at the nanometer level with the properties of the starting materials [34], making these materials appropriate for future technological applications. For this reason, they are usually considered to be innovative advanced materials, with promising applications in many fields, such as optics, biology, mechanics, sensors, electronics and coatings [54].

Hybrid properties strongly depend on the nature and strength of the interactions between the organic and inorganic components [55]. According to the classification of the hybrid materials proposed by Judenstein and Sanchez [56], based on the nature of their interactions, these materials have been divided in two classes.

**Figure 3:**
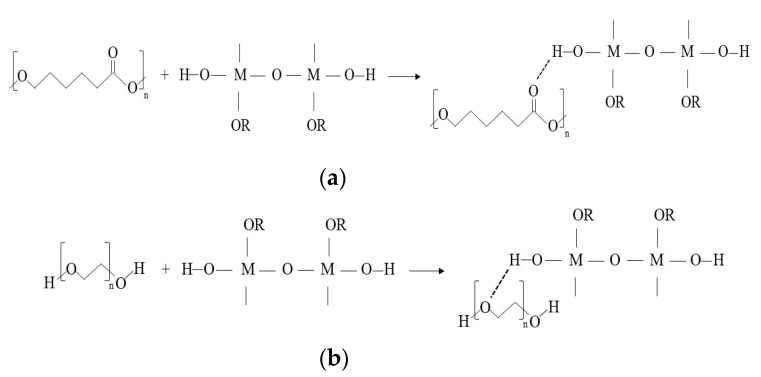
A graphical representation of the (**a**) synthesized glass/PCL and (**b**) synthesized glass/PEG hybrids.

In so-called “Class I” hybrids, the constituents are held together by weak forces such as hydrogen bonds or van der Waals, or π–π interactions. The hybrids in Class I can be produced using different techniques. In particular, Catauro et al. synthesized Class I hybrid materials with the sol-gel method [16,57,58], thus obtaining materials in which the organic and inorganic phases were linked by hydrogen bonds between the hydroxyl group of the inorganic matrix and the amide, carboxyl or carbonyl groups present in the polymer chains (Figure 3). Polymers like poly-ε-caprolactone (PCL) or polyethylene glycol (PEG) represented the organic components, while silicon, titanium and zirconium dioxides (SiO_2_, TiO_2_ and ZrO_2_, respectively) were considered as the inorganic matrixes.

Gill et al. and Wojcik et al. produced hybrids by simultaneous gelation of organic and inorganic components with interpenetrating inorganic and organic substance networks [59,60], thus obtaining elastic hydrogel, flexible rubber and hard glass with mesoporous networks accompanied by a high degree of biocompatibility [61].

The main characteristic of Class II hybrid materials is the presence of strong covalent or ionic-covalent bonding that connects the organic and inorganic components. This class of materials could be prepared by using a telechelic polymer such as hydroxyl-terminated poly(dimethylsiloxane) (PDMS) [62,63].

As stated above, hybrid materials can be used for a wide range of applications such as optics, electronics, ionics, mechanics, energy, environment, biology, and medicine, for example, as membranes and separation devices, fuel and solar cells, catalysts, sensors and functional smart coatings [47]. For this reason, one of the main aims of many studies is chemical characterization in order to elucidate the properties of inorganic and organic components in the view of their potential and actual applications.

### 3.1. Hybrid Materials: Characterization Performed According to a Multi-Technique Approach

Hybrid materials are highly dependent on composition, particle size and their interactions [64]; for this reason, a careful and accurate chemical, morphological and thermal characterization is necessary.

The techniques that satisfy this need include Fourier transform infrared (FTIR) spectroscopy and solid-state Nuclear Magnetic Resonance (NMR), as well as differential scanning calorimetry (DSC), differential thermal analysis (DTA), and thermogravimetry (TG) [57,65]. The FTIR bands of the hybrid materials usually show typical signals related to the M–O–M bonds found in the inorganic network and the typical bands of the bonds formed in the polymer chains. The intensity of the bands related to each phase is a function of their relative amount in the hybrids. Moreover, when the condensation reaction is not complete, the signal of the residual M–OH bonds in the inorganic matrix is still visible. FTIR spectra can also provide evidence of the presence of hydrogen or covalent bonds between the organic and inorganic components. Many studies report the formation of hydrogen bonds between the polymer and the residual M–OH bonds of the inorganic network in the hybrids, proved by FTIR spectra [66,67,68].

NMR solid-state spectra usually confirm the results derived by FTIR analysis and can provide further information on the structure of the metal oxide network and on its condensation rate. This technique requires special equipment, comprising magic-angle spinning (MAS), cross polarization (CP), etc. [69]. In the corresponding spectra, the signals related to the network forming the atomic nucleus are generally denoted by the Qn symbol to show the substitution degree of the structural units. For example, for a ^29^Si CP MAS NMR spectrum of a silica-based hybrid, n represents the number of bridging oxygen bonds formed by the SiO_4_ tetrahedral units. Therefore, a sample showing a chemical environment consisting of only Q4 would possess a full condensed metal oxide network phase; in a silica-based hybrid, this case corresponds to the stoichiometric SiO_2_ [70]. Conversely, the presence of Q3 and Q2 structures is a confirmation that an incomplete condensation has taken place.

The microstructure of the hybrid materials may be revealed by X-ray techniques and neutron scattering, whereas their morphology can be investigated using some microscopy techniques. In particular, X-ray diffraction (XRD), wide-angle X-ray diffraction (WAXD) or wide-angle X-ray scattering (WAXS), are widely used in the characterization of hybrid materials. The presence of crystalline phases in the materials, and eventually their identification, is detected by XRD [71], while the degree of crystallinity, an important parameter that remarkably influences the physical, mechanical and biological properties of materials, is analyzed by WAXD [72].

Transmission electron microscopy (TEM), scanning electron microscopy (SEM) and atomic force microscopy (AFM) are successfully used to closely study the morphology of the hybrids. In particular, TEM (based on the transmission of a beam of electrons through an ultrathin specimen) is able to provide information about the inner structure of the analyzed sample. When the samples interact weakly with the electron beam, stains such as phosphotungstic acid and RuO_4_ can be used to improve the quality of the recorded images. Sometimes, the energy of the electron beam can be responsible for the occurrence of the decomposition of the organic components of the hybrid. To overcome this drawback, cryogenic microscopy (cryo-TEM) can be used, where the sample is analyzed at liquid nitrogen or liquid helium temperatures in a frozen state [73].

SEM uses a source of electrons that, once emitted, strike the sample surface and are inelastically scattered by the atoms in the sample. Therefore, it allows 3D sample images to be recorded and, thus, can be successfully used to analyze the surface structure of the sample [74], with the detection of such emitted electrons being responsible for the formation of the sample image. Moreover, in addition to the emitted electrons, X-rays are also produced by the interaction of electrons with the sample. An SEM equipped for energy-dispersive X-ray (EDX) spectroscopy can provide sample surface microanalysis by detecting the emitted X-rays [75].

AFM is a microscopy technique that acquires information about the morphology of the sample surface. It consists of a sharp tip (10–20 nm diameter) attached to a stiff cantilever. The tip is brought as close as possible to the surface when the sample is scanned under the tip. The tip moves in response to a tip-surface interaction, and this movement is measured by focusing a laser beam onto the back of the cantilever, followed by the detection of the position of the reflected beam with a photodiode. AFM is an effective and widely used tool for characterizing nanocomposites [76,77].

X-ray photoelectron spectroscopy (XPS) is used to evaluate the surface compositions of a specimen, by treating the sample under high vacuum to X-rays, which penetrate (approximately some nanometers) into the top layer of the sample and excite the electrons. Some of these electrons from the upper layer are emitted from the sample and can be detected, provided that the electron binding energy is a function of the chemical environment of the atom. Therefore, it is possible to use the XPS technique with the aim of identifying the elements found on the sample surface of the organic–inorganic hybrids under investigation [78,79,80].

### 3.2. Hybrid Materials: Thermal Characterization

The structure (porosity, crystallinity degree, etc.), and thus the properties, of sol-gel materials are considerably influenced by the drying and heating conditions used for the post-synthesis treatments of the obtained gels. During those processes, polycondensation reactions take place, thus leading to the formation of further M–O–M bonds via the removal of –OH group residues in the gel network (dehydroxylation) [11]. In addition, the elimination of the water, solvents and organic modifiers (e.g., catalysts) takes place under isothermal superambient temperature or at ambient temperature under vacuum conditions, or upon heating at a relatively low temperature. Therefore, the thermal behavior with particular reference to drying and heating conditions (e.g., heat treatment temperature, heating rate, etc.) is preliminarily worth studying to obtain materials with a specific microstructure and, thus, definite properties.

Many studies have reported in the literature that the heating conditions (which generally result in different degrees of crystallization of the final materials) may affect optoelectronic [81], magnetic [82], and luminescence [83] properties, photocatalytic efficiencies [84], nanoparticle size [85], and many other features of the obtained sol-gel materials.

In particular, different degrees of crystallization extensively influence the biological performance of those materials [86,87,88,89]. Cell adhesion and material bioactivity, namely the ability to induce hydroxyapatite nucleation on their surface when soaked in a simulated body fluid [6], are strictly related to their osseointegration ability, influenced by surface topography and ion release capability [90,91,92], which, in turn, depend on the material structure.

Severe drying and heating conditions during the synthesis of organic–inorganic hybrid materials must be avoided, since they can cause the degradation of the organic component, irreversibly modifying their properties. Therefore, in-depth knowledge of the thermal behavior of sol-gel materials is essential, and is needed for the determination of the optimum conditions for the calcination of sol-gel materials [93,94,95,96,97]. This characterization can be effectively performed using thermal analysis techniques, which include differential scanning calorimetry (DSC), differential thermal analysis (DTA), thermogravimetry (TG), thermomechanical analysis (TMA), dynamic mechanical analysis/dynamic mechanical thermal analysis (DMA/DMTA), dielectric thermal analysis, etc. The change of the defined physico-chemical properties (namely, the heat flow, temperature and mass for DSC, DTA and TG, respectively) is recorded as a function of time and/or temperature when the sample is subjected in a condensed phase (usually solid in the form of a powder) to a temperature program (with isothermal, constant heating or cooling rate being the most commonly adopted) under a given inert or reacting flowing gas atmosphere.

In some cases, some techniques are used simultaneously (TG/DTA and TG/DSC) to collect supplementary information on the nature of the process occurring during the thermal analysis experiment: an endothermic sharp DTA or DSC peak with no mass loss detected in the TC curve suggests that melting probably takes place. Simultaneous TG/DTA and TG/DSC apparatuses are preferably used to determine the thermal properties of polymer nanocomposites. The comparison of both TG and DTG (the first-order derivative of TG data) curves makes it possible to more easily detect the onset degradation temperature, commonly used to assess a thermal stability scale in a series of materials undergoing the same process (described by the same reaction mechanism) [98,99,100,101]. DTA and DSC techniques can be efficiently used to determine (from a qualitative or quantitative point of view, respectively) the thermal effects due to physical or chemical processes (phase transitions or dehydration and thermal decomposition, respectively).

### 3.3. Hybrid Materials: Biomedical Applications

Hybrid materials have more and more applications in the biomedical field and are becoming increasingly important, thus increasing the replacement of our body parts. The sol-gel process offers new and interesting possibilities for encapsulating heat-sensitive and fragile biomolecules such as proteins, enzymes, and antibodies, because this route takes advantage of the inherent low temperature and biocompatible process [102]; however, in aqueous solutions, biomolecules lose their functionality. Therefore, this inconvenience can be minimized by direct immobilization of active biological substances in porous metal oxide carriers using sol-gel processes. This is possible due to its simplicity of preparation, low-temperature encapsulation, easy immobilization, chemical inertness, tunable porosity, optical transparency, mechanical stability and negligible swelling behavior [103,104].

Silica has been widely used as an inert and stable inorganic matrix for immobilization of different biological systems, particularly the immobilization of enzymes, antibodies and proteins. The silica loaded with biomolecules obtained using the sol-gel method has led to important biotechnological applications. Sanchez et al. [105] described the advantages of the silica matrix for enzyme immobilization, resulting from its high specific surface areas and controllable pore diameters, which can be tailored to the dimensions of a specific enzyme.

The sol-gel method presents a poor loading efficiency and enzyme leakage, which can be overcome by the design of specific protocols that pay attention to matrix preparation with a pore size that adequately allows the flowing of substrates and products, while at the same time being small enough to avoid the elution phenomenon of the entrapped biomolecules [106].

As with enzymes, proteins may also be encapsulated in silica materials obtained using the sol-gel method; indeed, they are able to reside in pores whose size is comparable to the size of the proteins. Thus, it is worth stressing that whether the native protein conformation still remains intact during and after the entrapment, the proteins are also able to change their conformation once entrapped. This is an important ability, because in many cases, the binding between the analyte and the protein induces conformational changes [107]. When the proteins are entrapped in the sol-gel matrix, they should remain functional, because their function depends mainly on their conformational structure. Das et al. [108] suggested that proteins tend to preserve their native conformation immediately upon the entrapment, although some of them (for example the myoglobin) may undergo substantial conformational changes during the process.

Another example of biomolecule encapsulation using the sol-gel method is that of antibodies, which are proteins whose molecular weight is very high. If the antigen that is the targeted antibody compound possesses a high molecular weight, the interactions between them could be very difficult, since complex formation is affected by the small pores of the matrix. Yang et al. [109] reported that there is no effect on antibody encapsulation efficiency as a result of the addition of PEG, but there is a strong effect on the binding activity of the encapsulated antibody.

The wide versatility of the sol-gel method enables the loading of antiproliferative, anti-migratory, anti-thrombotic, anti-inflammatory and anesthetic agents, anti-coagulants, cell growth promoters and inhibitors, and other pharmaceutical drugs in an inorganic matrix. Very few parameters (degree of porosity and the specific surface of the pores, the amount of the organic component, and aging/drying conditions) influence the incorporation of drugs [110].

An ideal drug delivery system is able to deliver the therapeutic drug amount to the proper target in the body, acting promptly, and hopefully maintaining the desired drug concentration and effect [111]. The strength of the interactions between drugs and the sol-gel material, given by the functional groups of the main component of the system [112], can influence the release rate. The study on the release kinetics of active compound could follow the Higuchi model [113,114], which can be used for both water and low-soluble drugs incorporated in solid/semi-solid matrixes obtained using sol-gel synthesis. If the matrix includes biodegradable polymers in biological medium, governed by the local enzymes, the drug release may occur via a degradation mechanism [115]. When the drug is released due to osmotic swelling, the kinetics release fits to Fick’s law. In this case, the water penetrates into the drug carrier system, inducing relaxation of the polymer chain, and thus the release of the drug outside. An increase in drug release can be obtained using porous materials obtained by controlled drying or by using a modified catalyst molar ratio during synthesis [116].

In recent decades, many newly designed drugs based on biomolecules such as peptides, proteins, and DNA have been considered. These molecules are chemically unstable, and thus encapsulation resulted in protecting them and making them available to deliver drug molecules to the target tissues [117]. Unfortunately, many cancer drugs have poor solubility and instability. Therefore, the development of delivery systems that can carry a high payload of drug, while protecting the drug from degradation, facilitating cellular uptake and targeting specific cell populations is necessary for their clinical applicability [118]. Among the anticancer drugs considered for this purpose, doxorubicin (DOX) was one of the more promising [119], since it was possible to deliver DOX to tumor sites and observed the apoptosis ex vivo in the tumor cells. Hillegass et al. [120] applied similar sol-gel silica-based nanoparticles as carriers for DOX to treat malignant mesothelioma. DOX-loaded and DOX-free nanoparticles were both injected subcutaneously and directly into the tumor or intraperitoneally in an in vivo mouse model.

Finally, sol-gel materials make it possible to prepare medical diagnostic tools that possess biocompatible, non-toxic and/or drug delivery properties [66]. Some results show that the use of polymers as substrates expands the properties of devices, since the polymeric moiety can hold the drug onto the surface of implantable medical devices, and exact drug release occurs via degradation of the polymer [121].

## 4. Preparation and Characterization Details of Hybrid Materials

### 4.1. Synthesis by Sol-Gel Route of Hybrid Materials

Hybrid materials are particularly interesting for application in the biomedical area. Catauro et al. prepared several hybrid materials using the sol-gel method, with different inorganic matrices and different polymers [16,57,58,89,95,97,122]. In particular, two different polymers, namely PCL or PEG, were incorporated at different concentrations into several oxides (acting as inorganic components): silicon, titanium and zirconium dioxides (SiO_2_, TiO_2_ or ZrO_2_). Worth mentioning are the results obtained with ZrO_2_/PEG hybrids [19,57], in which PEG was incorporated in increasing amounts (from 6 to 70 wt%) in the zirconia matrix.

**Figure 4:**
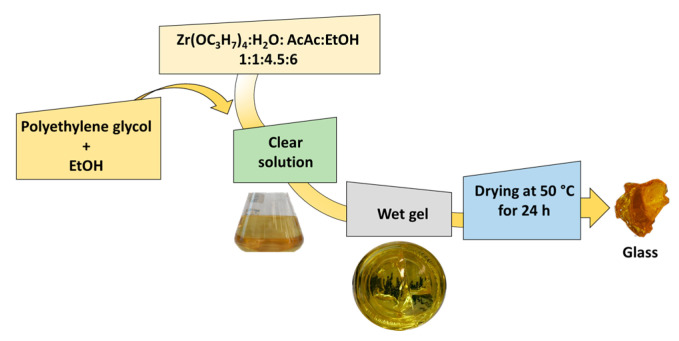
Synthesis by the sol-gel method of hybrid materials.

A zirconium (IV) propoxide, Zr(OC_3_H_7_)_4_, solution (70 wt% in n-propanol, supplied by Sigma Aldrich, St. Louis, MO, USA) and PEG (purchased from Sigma-Aldrich, St. Louis, MO, USA, with average molar mass of about 400 g mol^−1^) were used as precursors of the inorganic and organic phases, respectively. In the first step of the sol-gel synthesis, the precursor of zirconia was added to a mixture of ethanol, water and acetylacetone (AcAc, Sigma-Aldrich, St. Louis, MO, USA) with molar ratios Zr(OC_3_H_7_)_4_:H_2_O:AcAc EtOH = 1:1:4.5:6. AcAc was used to control the hydrolytic activity of zirconium alkoxide. Subsequently, PEG, previously dissolved in ethanol, was added to the solution under strong stirring to obtain a homogeneous sol. Subsequently to gelation, the wet gels were air-dried at 45 °C for 48 h to eliminate the solvent. Figure 4 shows the synthesis by the sol-gel method of hybrid materials.

**Figure 5:**
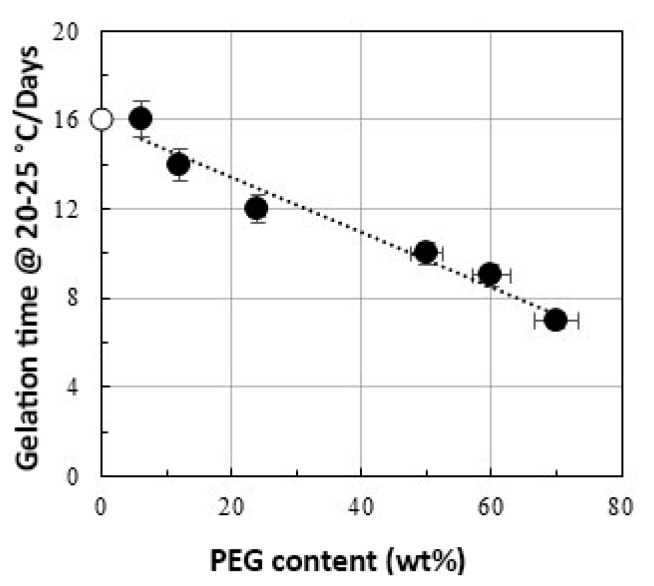
Dependence of the gelation time on the PEG content in the hybrid (expressed as wt%).

The gelation process is a mechanism that transforms a liquid solution of macromolecules or particles into an elastic well-shaped solid. Figure 5 shows the gelation time at 20–25 °C, which decreases almost linearly with the PEG content.

### 4.2. Coupling of FTIR and TG Measurements for the Assessment of Thermal Decomposition Steps

The thermal behavior of pure ZrO_2_, HAp and composites was studied by means of a simultaneous Mettler Toledo TG/DSC 2950 apparatus (Mettler Toledo, Columbus, OH, USA), and STARe software (Version 9.00, Schwerzenbach, Switzerland) was adopted to process the experimental data. The unit includes two crucibles, a reference one containing alumina powder, while the sample one was filled with about 20–25 mg of material with a view to entirely covering the bottom of the crucible. The TG measurements were performed under inert N_2_ purging gas at 60 mL min^–1^ up to 800 °C at 10 °C min^–1^. The calibration of temperature was performed using very pure calibrant substances (indium and zinc in this study with purity greater than 99.998%). As a consequence, a final average uncertainty u(T) = ±1K may be assumed over the temperature range investigated. To assess the reasonable mechanisms for each process occurring during the TG/DSC measurements, the gases evolved were detected using a SETARAM 92-16.18 TG apparatus (SETARAM, Caluire, France) coupled with a Thermofisher Scientific Nicolet iS10 Spectrophotometer (Thermofisher, Waltham, MA, USA). Alumina crucibles containing about 100–150 mg of sample were used to gather and detect the evolution of the minimum content of gases upon heating, investigated by FTIR using the Calisto software. Preliminarily, blank experiments were carried out with empty crucibles using the same experimental conditions: argon flow at 40 mL min^–1^, and temperature ranging from 25 and 1200 °C at 10 °C min^–1^. The gases produced during the TG experiments were collected in the FTIR apparatus via a transfer line heated at 200 °C to avoid the occurrence of condensation of any compound. A single spectrum was recorded every 11 s, with eight scans being carried out at 0.5 cm^–1^ intervals with a resolution of 4 cm^–1^. The results of TG and DTA experiments of pure ZrO_2_ and the six ZrO_2_/PEG hybrid materials are summarized in Table 2. All the materials first underwent a step of mass loss, attributed to dehydration. The highest amount of water was found in ZrO_2_, followed by the PEG-poor hybrids, ZrO_2_/PEG6% and ZrO_2_/PEG 12%, while lower content was found in the PEG-rich hybrids (ZrO_2_/PEG 24%, ZrO_2_/PEG 50%, ZrO_2_/PEG 60% and ZrO_2_/PEG 70%). Subsequently, dehydroxylation took place in ZrO_2_, with water lost through condensation of hydroxyl surface groups, as was recently ascribed to pure SiO_2_ [97]. Under inert purging gas, this process occurred slowly in a wider temperature range from 200 to about 600 °C [123]. Conversely, PEG decomposition took place in all ZrO_2_/PEG hybrids between 200 and 350 °C in a single step of mass loss. This process was simultaneously accompanied by some endo-exothermic reactions. In previous studies, the thermal degradation of pure PEG under inert atmosphere was found to occur at about 423 K [124]. The embedding of PEG into the ZrO_2_ matrix caused a mutual interaction responsible for a shift toward higher temperatures of PEG degradation.

The mechanism of thermal degradation involves pyrolysis reactions though a radical chain pathway initiated by random scissions of C–C or C–O bonds. After decomposition of PEG, a third step of mass loss takes place, with mass loss from about 7 (for ZrO_2_ sample) to 15 wt% (for sample ZrO_2_/PEG70%). The onset temperature is shifted toward lower values with increasing PEG content. The corresponding exothermic effect confirms that this mass loss step is not ascribable to PEG degradation, but to a decomposition/oxidation of AcAc [125], thus leading to the formation of both gaseous and non-volatile products. As a result, a slight mass loss is found in this range for two reasons: the sol-gel synthesis involves a low amount of AcAc, and a solid residue is formed. However, the higher the amount of PEG, the higher the mass loss recorded. When a metal coordination complex of AcAc is synthesized, the formation of its enol form is favored over that of diketone, resulting in the bonds between the –OH terminal groups of the PEG chains and the alpha carbons of the AcAc molecules [126]. Therefore, the thermal degradation of the thus-obtained PEG–AcAc compounds leads to the formation of products with a molecular weight higher than those formed in pure zirconia (PEG-free material, ZrO_2_). The exothermic effects observed in this temperature range upon heating are also attributable to the formation of tetragonal zirconia nanocrystals.

Moreover, with a view to establishing an eventual stabilizing effect with respect to PEG degradation due to the interaction between the polymer and the inorganic ZrO_2_ matrix, the onset degradation temperatures (T_onset_) of the ZrO_2_/PEG materials are also reported in Table 2. A correlation was found with the increasing content of PEG, similarly to what was recently determined in other PEG/clay composites [127].

**Figure 6:**
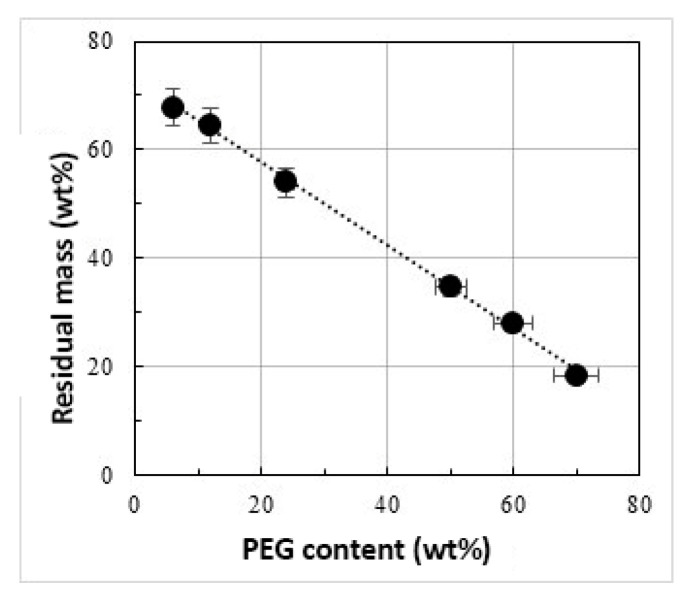
Linear decreasing trend of the sample mass of the residue after the TG experiments with increasing content of PEG in the hybrids.

Recently, a linear decreasing trend of the T_onset_ against PEG content was found in similar PEG-based organic–inorganic hybrids [97]. In fact, when a high amount of polymer is added into the sol-gel matrix, only a part of it may form hydrogen bonds with the matrix, since the number of –OH groups is too low to bond all of the PEG chains. As a consequence, the PEG moieties non-linked to the inorganic matrix are not influenced by the corresponding stabilizing effect, thus causing a decrease of the degradation temperature [97]. Figure 6 shows an evident linear decreasing trend of the sample mass of the residue after the TG experiments with increasing content of PEG in the hybrid materials.

For all PEG-based hybrids, a decreasing trend of the residual masses (ascribable to ZrO_2_) is found after the complete degradation of the polymer with increasing the PEG content, with the exception of PEG-free pure ZrO_2_ material.

Table 3 reports the wavelengths of the most significant bands related to the FTIR spectra of ZrO_2_/PEG, pure zirconia and PEG-based hybrid materials. As expected, the spectrum of ZrO_2_/PEG 6% (ZrO_2_/PEG system with 6 wt% of PEG) is quite similar to the spectrum of pure zirconia, because of the extremely low concentration of the polymer. On the other hand, the corresponding FTIR spectra become more similar to the spectrum of pure PEG as the content of PEG increases. In particular, the signal of the ethereal C–O–C stretching at 1104 cm^−1^ is already visible in ZrO_2_/PEG 12% and ZrO_2_/PEG 24%, while the C–H bending of the polymer at 1454 cm^−1^ [128] is clearly evident along with the bands attributed to alcohols (C–O stretching at 1250 cm^−1^) in the PEG-rich samples. In the spectra of ZrO_2_/PEG 60% and ZrO_2_/PEG 70% (containing 60 and 70 wt% of PEG, respectively), the intensities of the bands at 1585 and 1529 cm^−1^ decrease and appear slightly up-shifted compared with that of the spectrum of ZrO_2_/PEG 50%. This observation was ascribed to the formation of interactions between PEG and AcAc, which increases with the polymer content. Furthermore, the formation of H bonds between the hydroxyl group of the inorganic matrix and the PEG chains is suggested by the change in the shape of the band assigned to Zr–OH stretching at 654 cm^−1^ and of the broader band of the –OH at 3440 cm^−1^ [95]. Finally, the peaks at 460 and 422 cm^−1^ are assigned to Zr–O–Zr stretching and Zr–O–AcAc vibrations [57]. For these reasons, the materials prepared in this study are classified as class I hybrids [56].

The ^1^H–^13^C CPMAS NMR spectra of the hybrids confirms the findings derived by FTIR ones [57]. In particular, the small shoulder at about 72 ppm is due to the weak interactions between the carbon chain and the inorganic component, while the peak at about 71 ppm is attributed to the ether carbons belonging to the polymer chains, and the smaller peak at 61 ppm is due to the carbons connected to the –OH terminal groups.

### 4.3. Structural and Morphological Characterization

The XRD spectra of both the pure zirconia and the ZrO_2_/PEG 70% exhibit a broad hump, which is characteristic of amorphous materials, while no peak related to the crystalline phase of PEG is clearly visible in the spectra of all the hybrid materials, regardless of the amount of polymer [57,129].

The SEM micrographs of the cross section of the hybrid materials show that the PEG-poor hybrids (ZrO_2_/PEG 6%, ZrO_2_/PEG 12%, ZrO_2_/PEG 24%) and pure zirconia had comparable morphologies and are homogenous materials (Figure 6). On the other hand, the PEG-rich samples, namely ZrO_2_/PEG 50% and ZrO_2_/PEG 70%, were different from those with low content of PEG: the fiber-like structures disappear, and they appear very rough, thus suggesting that modulation of the polymer content could be a tool for modifying the microstructure of such hybrids.

**Figure 7:**
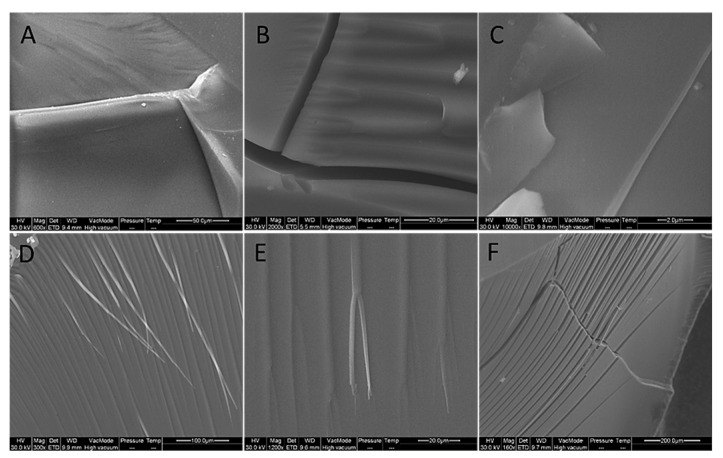
SEM micrograph of sample surface: (**A**) ZrO_2_, (**B**) ZrO_2/_PEG 6%, (**C**) ZrO_2_/PEG 12%, (**D**) ZrO_2_/PEG 24%, (**E**) ZrO_2_/PEG 50%, (**F**) ZrO_2_/PEG 70%.

However, Figure 7 shows that no phase separation is visible, and both kinds of sample appear homogeneous.

### 4.4. Bioactivity Tests

All composite materials were crushed to obtain a powder using an agate mortar, and they were soaked for 7, 14 and 21 days in a simulated body fluid (SBF) with an ion concentration nearly equal to that in human blood plasma, as suggested by Kokubo.

The SBF solution was exchanged every 2 days to avoid depletion of the ionic species in the SBF due to the formation of biominerals. After 7, 14 and 21 days of exposure at 37 °C, the samples were removed from the SBF and were air-dried in a desiccator. To evaluate the in vitro bioactivity of the samples, the formation of hydroxyapatite on the materials surface was detected by SEM/EDX microscopy observations of the materials after soaking in SBF [6,19].

**Figure 8:**
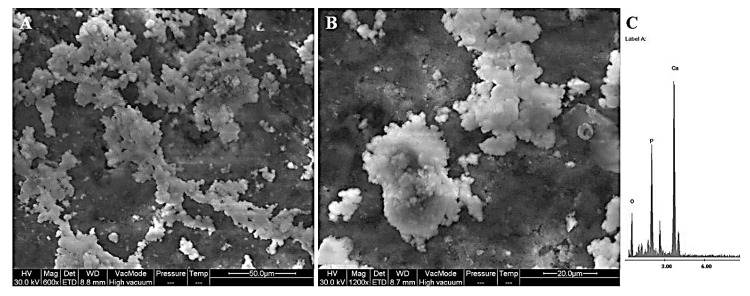
SEM images of samples after soaking in SBF for (**A**,**B**) 21 days. (**C**) EDX analysis.

After 21 days of incubation, the morphology of all the hybrid materials was studied with SEM, the images of which displayed in Figure 8 reveal that they exhibit a similar deposition of apatite on their surface. The EDX analysis of the crystals showed an atomic Ca/P ratio equal to 1:6, which is in good agreement with the chemical formula of hydroxyapatite [Ca_10_(PO_4_)_6_(OH)_2_]. The hydroxyapatite formed on the materials can be explained by the presence of surface Zr-OH groups on the samples, which interact with the Ca^2+^ ions present in the SBF. The Ca^2+^ ions combine with the negative charge of the phosphate ions to form amorphous phosphate, which is spontaneously converted into hydroxyapatite. These results suggest that the ZrO_2_/PEG samples are bioactive, regardless of the polymer content.

### 4.5. Antiradical Capacity

The biocompatibility tests of the hybrid materials obtained using the sol-gel technique were carried out using the NIH–3T3 murine fibroblast cell line using the conventional MTT assay. Cell viability was expressed as the percentage of mitochondrial redox activity of the cells treated with the materials compared to an untreated control. Fibroblasts were grown for 24 and 48 h in the absence and presence of pure ZrO_2_ and ZrO_2_/PEG hybrid materials, each containing different percentages of the polymer [19]. The results show that all the hybrids tested were able to induce an increase in cell viability compared to what was observed in pure zirconia when it was tested in non-hybrid form. The data made it possible to observe that the effects were dose- and time-dependent. The presence of different amounts of polyethylene glycol promoted the preparation of biomaterials with improved biocompatibility.

### 4.6. Antibacterial Activity

**Figure 9:**
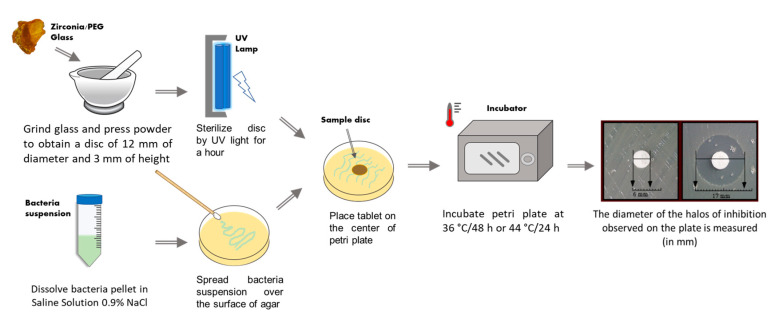
Agar diffusion test used for evaluating antimicrobial activity of ZrO_2_/PEG hybrid material.

Zirconia-based materials have been reported to be effective as antibacterial agents, because they can interact with bacteria without penetrating into the cell [130,131,132]. The agar diffusion test, the primary method for evaluating the antimicrobial activity of biomaterials, was carried out to obtain preliminary information on ZrO_2_ and ZrO_2_/PEG hybrids. For the test, two Gram-negative bacterial strains, *Escherichia coli* (ATCC 25922)(KWIK-STIK, US-MN), and *Pseudomonas aeruginosa* (ATCC27853) (KWIK-STIK, US-MN), and a Gram-positive bacterial strain, *Staphylococcus aureus* (ATCC 25923) (KWIK-STIK, US-MN), were used. The flowchart in Figure 9 is representative of the antimicrobial test used. ZrO_2_/PEG hybrids were crushed into a powder, weighed to obtain 100 mg of each sample tablet, and sterilized for an hour with UV light before the test. A pellet of bacteria strain was solubilized in 3 mL of saline water (0.9 % NaCl) to obtain a suspension of 10^−5^ CFU/mL. *E. coli* was grown on TBX Medium (Tryptone Bile X-Gluc), *P. aeruginosa* was cultured in Pseudomonas CN Agar (Liofilchem, Roseto degli Abruzzi (TE Italy), while *S. aureus* was cultured on Baird-Parker agar (Liofilchem, Roseto degli Abruzzi (TE), Italy). All media were sterilized at 120 °C for 15 min. After the sterilization process, the Baird-Parker agar was cooled to 50 °C and an emulsion of egg yolk containing potassium tellurite was added. A CN pseudomonas supplement was added to Pseudomonas CN Agar when it was cooled to 50/45 °C. After plating the bacteria suspension on all agar surfaces, the powder was placed on the center of the plate; *E. coli* was incubated at 44 °C for 24 h, while *P. aeruginosa* and *S. aureus* were incubated at 36 °C for 48 and 24 h, respectively. The diameter of the inhibition halos (*IDs*) was calculated in relation to the Petri Plate diameter (*PPD*) (6 cm). Four measurements were carried out for each sample to determine the standard deviation of the mean. The results are expressed as Bacterial Viability (*BV*) (in percentage).

*BV* = ((*PPD* – *IDs*))/*PPD* × 100 (4)

(The bacterial viability without samples is expressed as 100% *BV*. The standard deviation of the mean is expressed as Relative Standard Deviation (RSD).

**Figure 10:**
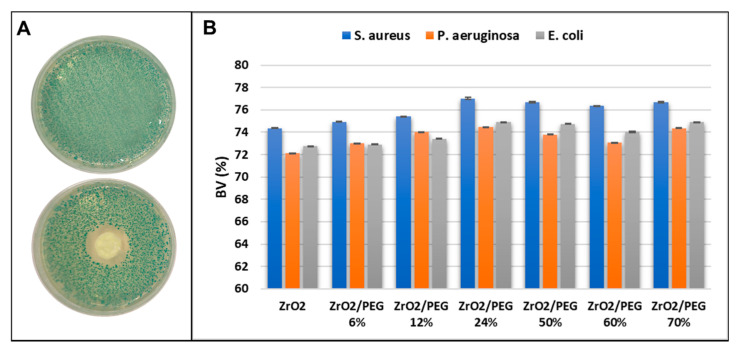
(**A**) Representative images of negative control and inhibition halo (ID) of *E. coli* with ZrO_2_/PEG 24%. (**B**) Comparison of bacterial viability of *S. aureus*, *P. aeruginosa* and *E. coli* in the presence of ZrO_2_/PEG hybrid materials. Bacterial viability without samples is expressed as 100%. The mean standard deviation is expressed as Relative Standard Deviation (RSD).

For antibacterial analysis, three different strains of bacteria were chosen to obtain preliminary information on the antimicrobial activity of these materials. Figure 10A presents an example of the bacterial inhibition in the presence of the ZrO_2_/PEG hybrid, and it is possible notice that the sample, placed onto the middle of plate, causes a slight inhibition of *E. coli* growth. Comparing the bacterial viability of the three bacteria strains in Figure 10B, it can be observed that the increase of PEG amount in the hybrid system does not influence bacteria inhibition, which is mainly caused by ZrO_2_. Figure 10B also reveals that *E. coli* and *P. aeruginosa*, Gram-negative strains, seem to be affected more than *S. aureus*.

## 5. Conclusions

The sol-gel technique has proved to be a valuable and versatile method for preparing organic–inorganic hybrid materials. Such synthesis offers considerable advantages such as high purity, mild processing conditions (atmospheric pressure and low temperature), homogeneity of products, low temperatures and the possibility of modifying the process conditions, with the aim of achieving tunable morphology, size and multifunctional properties strictly related to the nature of the interactions between the two components. New and interesting possible routes could be offered by this method especially when particular molecules such as proteins, enzymes, and antibodies can be encapsulated due to the low temperature. To not lose their functionality a direct immobilization of active biological substances is usually carried out in porous metal oxide carriers.

The materials synthesized and presented as a case study showed quite good qualities, thus demonstrating usefulness in the biomedical field. Spectroscopic techniques (FT-IR and solid-state NMR), XRD, and SEM microscopy confirmed that amorphous class I organic–inorganic hybrid ZrO_2_/PEG materials had been obtained. Indeed, FTIR and solid-state NMR ascertained that (PEG) was incorporated into the inorganic ZrO_2_ network by weak bonds (likely H-bonds) between the organic polymer and the hydroxyl groups of the inorganic matrix.

Moreover, the formation of a layer of hydroxyapatite on the surface after soaking samples in SBF, crucial for in vivo implant osteointegration shown by SEM and related EDX, indicated that the ZrO_2_/PEG materials were bioactive.

Comparing the bacterial viability of three bacterial strains, we were able to observe that the increase of PEG amount in the hybrid system did not influence bacteria inhibition, caused mainly by ZrO_2_, revealing that *E. coli* and *P. aeruginosa*, Gram-negative strains, seemed to be affected more than *S. aureus*.

The presence of different amounts of PEG in the hybrid materials indicated a positive behavior on cell growth and proliferation of NIH 3T3 cells. Their effects were greater than those exerted by pure ZrO_2_, ceramics from which are already widely used in joint replacements due to the strict relation between cell proliferation and the polymer content in the several hybrids. These results suggest that the incorporation of the polymer in the inorganic matrix favors the preparation of biomaterials with improved biocompatibility.

## Figures and Tables

**Table 1:** Example of applications of hybrid materials obtained via the sol-gel route.

Organic-Inorganic Hybrids from Sol-Gel	Properties	Application	Authors
Silica/Chitosan/HRP	Spherical particles 100–200 nm diameter High porosity	Biosensor	G.-H. Wang and L.-M. Zhang, 2006 [37]
Silica/Chitosan	Macroporous scaffold 100–500 µm pore size	Bone tissue engineering	Reis et al., 2007 [38]
ZrO_2_/PEG/Eu^3+^ (4.5 wt%)/ZnS (2–3.5 wt%)	Amorphous nanocrystals 12 nm	Optical devices	Jose et al., 2021 [39]
PU/HBNPSi	Homogeneous structure	Flame retardant	Shen et al., 2021 [40]
HMSs (hollow mesoporous silica spheres)	Spherical particles (400–421 nm) 2.8–3.9 nm	Cu^2+^ adsorption and electrostatic interaction	Sun et al., 2020 [41]
Silica/Starch/PEO or Calcium alginate	Matrix	Bioencapsulation of microbial cells	Evstatieva et al., 2014 [42]
Alcohol-Aminosilicate	Matrix	Anticorrosion	Figueira et al., 2014 [43]
PLGA/mesoporous silica	Microspheres (20 µm)	Drug delivery	Xue and Shi, 2004 [44]

**Table 2:** TG parameters (namely, temperature ranges, onset temperatures and mass loss percentages) corresponding to dehydration and decomposition processes.

	Dehydration			Decomposition		
Material	Δ*T* (°C)	*T*_on_ (°C)	Δ*m* (%)	Δ*T* (°C)	*T*_on_ (°C)	Δ*m* (%)
ZrO_2_	15–200 200–400 ^1^	82 255 ^1^	19.7 9.9 ^1^	400–600	461	6.6
ZrO_2_/PEG6%	16–191	79	13.8	200–350 400–600	299 488	16.9 8.2
ZrO_2_/PEG12%	15–188	77	13.5	200–350 400–600	296 491	19.0 8.1
ZrO_2_/PEG24%	15–189	74	11.4	200–350	291	28.3
ZrO_2_/PEG50%	16–188	74	11.5	200–350	287	40.8
ZrO_2_/PEG60%	15–195	72	11.7	200–350	274	48.5
ZrO_2_/PEG70%	16–188	81	11.6	200–350	278	56.1

^1^ Data refer to the loss of water due to condensation of hydroxyl groups (dehydroxylation).

**Table 3:** Characteristic FTIR bands for the investigated materials.

Wavelenght (cm^−1^)	Attribution of Each FTIR Band
3440	–OH stretching
2870	C–H stretching of PEG
1585 and 1377	C=O vibrations of AcAc
1529 and 1280	C–C vibrations
1454	C–H asymmetric bending
1425	C–H symmetric bending
1250	Alcohol C–O stretching
1104	Ethereal C–O–C stretching
1026 and 931	C–C–H bending and C–C stretching
654	Zr–OH stretching
460	Zr–O–Zr stretching
422	Zr–O–AcAc vibrations
